# Prognostic Impact of Baseline Potassium Abnormalities in Patients with Heart Failure with Reduced Ejection Fraction: A Real-World Cohort Study

**DOI:** 10.3390/medsci14030402

**Published:** 2026-07-18

**Authors:** Alvaro Roldan-Guerra, Jorge Perea-Armijo, Rafael González-Manzanares, Juan Carlos Castillo-Domínguez, Manuel Crespin-Crespin, Manuel Pan-Álvarez Ossorio, Manuel Anguita-Sánchez, José López-Aguilera

**Affiliations:** 1 Heart Failure Unit, Cardiology Service, Reina Sofia University Hospital, 14004 Cordoba, Spain; alvaroroldan97@gmail.com (A.R.-G.); rafaelglezm@gmail.com (R.G.-M.); juancarlos.castillo@quironsalud.es (J.C.C.-D.); crespin2@hotmail.com (M.C.-C.); manuelpanalvarez@gmail.com (M.P.-Á.O.); manuelanguita@secardiologia.es (M.A.-S.); mircardjla@gmail.com (J.L.-A.); 2 Instituto de Investigación Biomédica de Córdoba, IMIBIC (Instituto Maimonides de Investigacion Biomedica de Cordoba), 14004 Cordoba, Spain

**Keywords:** potassium abnormalities, hypokalaemia, hyperkalaemia, HFrEF, prognosis

## Abstract

Introduction: Abnormal serum potassium levels are a common issue in patients with heart failure with reduced ejection fraction (HFrEF), limiting the use of prognostically beneficial therapies. The primary aim of this study was to characterise the HFrEF population according to baseline potassium levels and to assess their impact on medium- to long-term prognosis. Methods: A retrospective study was conducted on a cohort of consecutive patients with HFrEF recruited at our centre. Patients were classified according to baseline serum potassium levels into: Group 1 (<3.5 mEq/L), Group 2 (3.5–5.0 mEq/L), and Group 3 (>5.0 mEq/L). Prognostic impact was assessed in terms of HF readmissions, HF mortality and all-cause mortality. Results: A total of 409 patients were analysed. The median age was 69 years [IQR 59–77], with a predominance of male patients (74.1%). There were 26 patients (6.36%) with hypokalaemia, 365 (89.24%) with normokalaemia, and 18 (4.4%) with hyperkalaemia. Group 1 showed a higher prevalence of diabetes mellitus (69.2% vs. 47.4% vs. 33.4%; *p* = 0.043) and a greater proportion of patients with ≥2 previous admissions for HF (25% vs. 10.5% vs. 0%; *p* = 0.008). No differences were observed regarding echocardiographic parameters, markers of congestion, or HFrEF treatment. After a median follow-up of 60 months, no significant differences were found between groups in rates of HF readmissions, HF mortality and all-cause mortality. Conclusions: Patients with hypokalaemia exhibited a higher prevalence of diabetes mellitus and previous HF admissions. In our population, no differences were observed between groups in the use of prognostically beneficial therapies or in outcomes in terms of HF rehospitalisations and mortality.

## 1. Introduction

Heart failure with reduced ejection fraction (HFrEF) is commonly associated with abnormalities in serum potassium levels, representing a highly relevant clinical issue. Approximately 18% of patients with chronic heart failure present with potassium disturbances, and up to 45% will experience such abnormalities at some point during the natural course of their disease [1,2]. In the setting of HFrEF, most available evidence is derived from clinical trials, so there is limited information regarding the prognostic impact of baseline potassium abnormalities in contemporary real-world cohorts of patients with HFrEF [2].

These abnormalities arise from the pathophysiological mechanisms inherent to heart failure (HF), the influence of various comorbidities, particularly chronic kidney disease (CKD) and diabetes mellitus (DM), diuretics and disease-modifying therapies for HFrEF, such as angiotensin-converting enzyme inhibitors (ACEi), angiotensin II receptor blockers (ARBs), and mineralocorticoid receptor antagonists (MRA) [3]. In contrast, sodium–glucose cotransporter 2 inhibitors (SGLT2i) have been shown to reduce the incidence of severe hyperkalaemia, while angiotensin receptor–neprilysin inhibitor (ARNI) are associated with lower rates of hyperkalaemia compared with ACEi and ARB [4]. Furthermore, the frequent episodes of decompensation that characterise the progressive course of HF together with the resulting hospital admissions, represent particularly vulnerable periods for the development of potassium disturbances.

The clinical relevance of these abnormalities lies in their potential to limit the use of therapies with established prognostic benefit in HFrEF, as well as in their apparent association with poorer outcomes in this patient population, primarily owing to potentially fatal arrhythmic events [5]. In patients with HF, mortality has been shown to follow a U-shaped relationship with serum potassium levels, although the optimal potassium range varies across studies and appears to be narrower than the reference ranges commonly used in routine clinical practice [6].

However, findings across studies remain inconsistent, and it is unclear whether the adverse prognosis associated with abnormal potassium levels reflects a causal relationship or merely a marker of increased risk [6]. Hypokalaemia has been associated with worse long-term outcomes, whereas hyperkalaemia appears to be associated with poorer short-term prognosis but not with long-term outcomes in HFrEF. Moreover, no clear relationship has been demonstrated between potassium abnormalities and HF rehospitalisation [2,7], further contributing to uncertainty in this area.

In recent years, new therapeutic strategies for the prevention of hyperkalaemia have been introduced, including well-tolerated potassium-binding agents such as sodium zirconium cyclosilicate and patiromer [3]. These therapies facilitate the appropriate titration of prognostically beneficial treatments [8,9], potentially improving outcomes, although they have not been shown to reduce mortality independently in this setting.

Accordingly, the present study was designed with the primary objective of evaluating, in a real-world cohort of patients with HFrEF, the medium- to long-term prognostic impact of baseline serum potassium levels in terms of HF readmissions, HF mortality and all-cause mortality. Secondary objectives were to assess the clinical characteristics of patients according to baseline potassium levels, compare optimisation of neurohormonal blockade therapy, and evaluate its impact on ventricular remodelling.

## 2. Materials and Methods

### 2.1. Study Design and Population

This was a single-centre, retrospective, longitudinal, observational study reflecting real-world clinical practice, which included all patients diagnosed with HFrEF who were consecutively attended to following either hospital admission or outpatient heart failure consultation between January 2018 and September 2020.

A total of 409 patients were included and classified according to baseline serum potassium levels: Group 1, patients with hypokalaemia (K^+^ < 3.5 mEq/L); Group 2, patients with normokalaemia (K^+^ 3.5–5.0 mEq/L); and Group 3, patients with hyperkalaemia (K^+^ > 5.0 mEq/L).

### 2.2. Clinical and Laboratory Variables

The aetiology of heart failure was established according to clinical criteria and the results of complementary investigations. Heart failure symptoms were defined according to the New York Heart Association (NYHA) functional classification. The number of previous heart failure admissions was defined as any hospital admission for heart failure requiring intravenous diuretic and/or inotropic therapy from the time of HFrEF diagnosis to the start of follow-up. Comorbidities were recorded based on clinical history at baseline.

Baseline and end-of-follow-up laboratory parameters were analysed. Anaemia was defined as haemoglobin levels <13 g/dL in men and <12 g/dL in women. Chronic kidney disease (CKD) was defined according to KDIGO guidelines [10].

Treatment-related data at baseline and at the end of follow-up were also analysed. The use of guideline-directed medical therapy for HFrEF was recorded, including ACEi, ARB, ARNI, β-blockers, MRA, ivabradine, loop diuretics, thiazides, and SGLT2i. In addition, dosage information was collected for diuretics and selected prognostically beneficial drug classes in this condition (Table S1).

Data on non-pharmacological therapies for HFrEF were also recorded, including cardiac resynchronisation therapy (CRT), defined as implantation of a resynchronisation device and physiological pacing via His-bundle or left bundle branch pacing, implantable cardioverter–defibrillator (ICD) therapy, and percutaneous treatment of mitral regurgitation using a transcatheter edge-to-edge repair device.

### 2.3. Echocardiographic Variables

Baseline and last follow-up echocardiographic data were analysed. The following variables of interest were collected: left ventricular end-diastolic volume (LVEDV, mL), left ventricular end-systolic volume (LVESV, mL), left ventricular end-diastolic diameter (LVEDD, mm), left ventricular end-systolic diameter (LVESD, mm), and left ventricular ejection fraction (LVEF, %), determined using the Teicholz or Simpson methods.

The presence of severe mitral regurgitation was also recorded, as assessed by quantitative and qualitative echocardiographic methods.

Improvement in LVEF during follow-up was defined according to the 2021 Universal Definition of Heart Failure [11].

### 2.4. Outcome Variables

Prognosis across groups according to baseline potassium levels was assessed in terms of HF readmissions, HF mortality, and all-cause mortality. Heart failure mortality was defined as death resulting from worsening heart failure (WHF) or sudden cardiac death. Unwitnessed or deaths of unknown cause were not considered cardiac-related in patients with life-threatening conditions such as active malignancy or severe infections. We compared the causes of death across the three study groups, categorising deaths as heart failure-related, sudden cardiac death, or non-cardiac. Recurrent heart failure readmissions were evaluated by comparing both the number of readmissions and their underlying causes across the three study groups.

### 2.5. Statistical Analysis

Continuous variables were expressed as mean and standard deviation. When the distribution deviated from normality, they were expressed as median and interquartile range. Categorical variables were expressed as absolute frequencies and percentages.

Normality was assessed using the Kolmogorov–Smirnov test and Q–Q plots. Comparisons between groups for categorical variables were performed using the Chi-squared test or Fisher’s exact test when appropriate, as well as McNemar’s test for paired data.

Comparisons between categorical and continuous variables were performed using ANOVA for parametric data or the Kruskal–Wallis test for non-parametric data. For within-group paired comparisons (before–after), repeated-measures ANOVA was used for parametric data or the Friedman test for non-parametric data. A univariate logistic regression analysis was performed to identify the variables associated with readmission and mortality due to heart failure.

For the baseline potassium category, patients with hypokalaemia and hyperkalaemia were each compared with the normokalaemia group, which served as the reference category. Variables with a *p*-value < 0.05 in the univariable analysis were subsequently included in a multivariable logistic regression model to identify independent predictors of heart failure readmission and all-cause mortality. Results are presented as odds ratios (ORs) with their corresponding 95% confidence intervals (95% CIs) and *p*-values.

Event-free survival (HF readmissions, HF mortality, and all-cause mortality) across groups and according to quartiles of baseline potassium distribution was analysed using the Kaplan–Meier method. Differences between survival curves were assessed using the log-rank test.

All tests were two-sided, and *p* ≤ 0.05 was considered statistically significant. All statistical analyses were performed using IBM SPSS software (version 25.0 for Macintosh, SPSS Corp., Armonk, NY, USA) and R version 4.5.1 (The R Foundation, Vienna, Austria).

### 2.6. Ethical Considerations

The study was approved by the Provincial Research Ethics Committee of Córdoba. The study complied with Good Clinical Practice standards and adhered at all times to the ethical principles outlined in the Declaration of Helsinki, including its most recent amendments and the Oviedo Convention. Data confidentiality was strictly maintained through anonymisation of the database, in accordance with Royal Decree 1720/2007, which implements Organic Law 15/1999 of 13 December on the Protection of Personal Data.

## 3. Results

### 3.1. Baseline Characteristics and Comorbidities

A total of 409 patients diagnosed with HFrEF were included in the study. The median age was 69 years (IQR 59–77), and the majority were male (74.1%). According to serum potassium levels at the start of follow-up, 26 patients (6.36%) were classified into Group 1, 365 (89.24%) into Group 2, and 18 (4.4%) into Group 3.

Baseline demographic and clinical characteristics are summarised in Table 1. No significant differences were observed in age or sex among the groups. Regarding comorbidities, patients in Group 1 had a higher prevalence of diabetes mellitus (69.2% vs. 47.4% vs. 33.3%; *p* = 0.043), whereas no significant differences were found for other comorbid conditions. In addition, patients with normokalaemia showed a non-significant trend towards a lower prevalence of CKD (61.5% vs. 39.7% vs. 50%; *p* = 0.07), with no differences in CKD stage according to estimated glomerular filtration rate.

Group 1 patients had a higher proportion of ≥2 heart failure admissions prior to study entry (25% vs. 10.5% vs. 0%; *p* = 0.008). However, no differences were identified regarding the proportion of de novo HF, duration of HF, aetiology, or functional class.

### 3.2. Electrocardiographic Parameters

Electrocardiographic findings are shown in Table 1. There were no differences in the prevalence of a wide QRS complex among the groups. However, complete left bundle branch block (LBBB) was more frequent in Group 1 (46.2% vs. 32.0% vs. 30.8%; *p* < 0.001), and this difference persisted at the end of follow-up (40.0% vs. 26.4% vs. 14.3%; *p* = 0.009).

### 3.3. Laboratory Parameters

Laboratory findings are summarised in Table 2. No differences were observed in serum creatinine, haemoglobin, electrolytes, or congestion biomarkers (NT-proBNP and CA125).

Regarding serum potassium levels at the end of follow-up, no statistically significant differences were observed (4.7 vs. 4.5 vs. 4.9 mEq/L; *p* = 0.29). Twelve patients (3.1%) had hypokalaemia, 361 (92.1%) had normal potassium levels, and 19 (4.8%) had hyperkalaemia. Furthermore, most patients in Group 1 achieved normal potassium levels by the end of follow-up (83.3%) (baseline median 3.2 mEq/L [3.1–3.3] vs. follow-up median 4.7 mEq/L [3.9–5.2]; *p* < 0.001). In Group 2, 2.9% developed hypokalaemia and 4.0% hyperkalaemia (baseline median 4.4 mEq/L [4.0–4.7] vs. follow-up median 4.5 mEq/L [4.2–4.9]; *p* < 0.001). Finally, in Group 3, 83.3% achieved normokalaemia at follow-up, whereas hyperkalaemia persisted in the remainder (baseline median 5.7 mEq/L [5.5–5.8] vs. follow-up median 4.9 mEq/L [4.2–5.4]; *p* < 0.001).

### 3.4. Echocardiographic Parameters

No differences were found in baseline echocardiographic variables among the study groups (Table 2). At the end of follow-up, improvements in LVEF and cardiac remodelling were observed across all groups, with no significant differences between them (Table 2).

The prevalence of severe mitral regurgitation was also similar at baseline. However, a significant reduction was observed in Group 2 (10.9% vs. 6.1%; *p* = 0.03).

### 3.5. Optimisation of HFrEF Therapy

With regard to treatment, no significant differences were identified among groups in baseline use of guideline-directed medical therapies with prognostic benefit in HFrEF. At the end of follow-up, there was a significant increase in the use of ARNI (34.2% vs. 59.4%; *p* < 0.001), SGLT2i (18.6% vs. 37.7%; *p* < 0.001), cardiac implantable electronic devices (CRT: 9.0% vs. 16.9%, *p* < 0.001; ICD: 4.6% vs. 17.8%, *p* < 0.001), and transcatheter edge-to-edge mitral valve repair (0.2% vs. 3.7%; *p* < 0.001). In addition, there was a reduction in the use of β-blockers (91.4% vs. 87.3%; *p* = 0.015) and loop diuretics (80.2% vs. 71.6%; *p* = 0.001), whereas MRA prescription remained unchanged (Table S2).

No differences were observed in the need for urgent haemodialysis. In all 11 events, the indication was volume overload, and in no case was hyperkalaemia the reason for dialysis initiation.

Between-group analyses showed that patients in Group 1 did not experience a reduction in loop diuretic use by the end of follow-up (88.5% vs. 96.2%; *p* = 0.50), unlike Groups 2 and 3. Indeed, Group 1 had a significantly higher proportion of patients receiving loop diuretics at the end of follow-up compared with the other groups (96.2% vs. 70.4% vs. 61.1%; *p* = 0.011). For all other therapies, the trends were similar to those observed in the overall cohort, with no differences in final treatment prescription among the groups.

Regarding the pharmacological titration, at baseline, patients in Group 1 had a higher proportion of low-dose ARNI therapy compared with the other cohorts (100% vs. 67.7% vs. 37.5%; *p* = 0.038), whereas no between-group differences were observed in the baseline dose titration of the other medications (Table S3).

During follow-up, only Group 2 showed a significant increase in the proportion of patients achieving high doses of ARNI (8.1% vs. 32.4%; *p* < 0.001), β-blockers (9.0% vs. 17.3%; *p* < 0.001), and MRAs (4.8% vs. 9.9%; *p* = 0.041). This pattern was not observed in the other cohorts, with the exception of Group 1, which also showed an increase in the proportion of patients receiving high-dose β-blockers (4.3% vs. 13.6%; *p* = 0.031).

However, by the end of follow-up, no between-group differences were observed in the dose distribution of any of the study medications. Likewise, there were no differences in the proportion of patients receiving monotherapy or dual therapy compared with those receiving triple or quadruple therapy (Table S3).

### 3.6. Prognostic Impact: Readmissions for Heart Failure and Mortality

**Figure 1:**
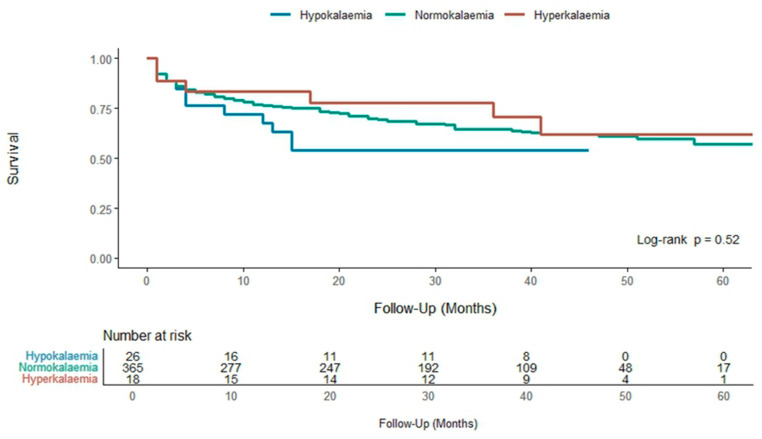
Survival analysis of time to readmission for heart failure according to baseline potassium levels.

**Figure 2:**
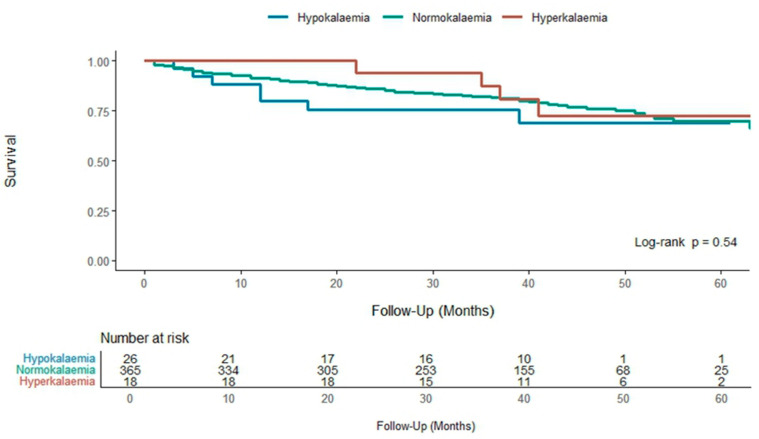
Survival analysis of time to death from heart failure according to baseline potassium levels.

**Figure 3:**
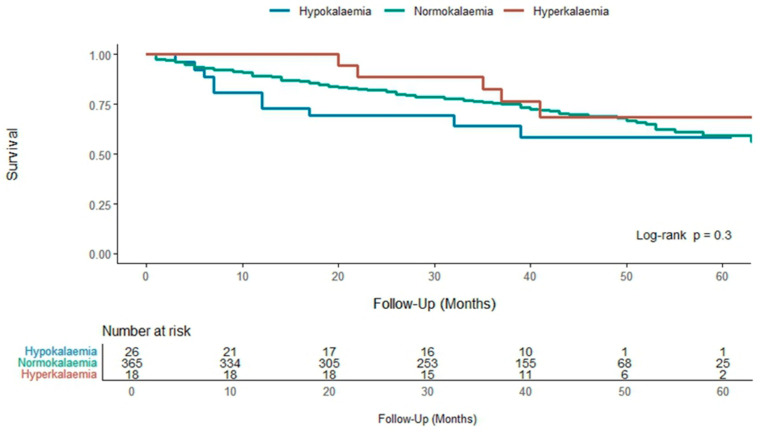
Survival analysis of time to death from any cause according to baseline potassium levels.

With a median follow-up of 60 months, there were no differences in the rate of readmissions for heart failure (46% vs. 42.9% vs. 38.1%; *p* = 0.517) (Figure 1) or in mortality from HF (31.2% vs. 30.4% vs. 27.4%; *p* = 0.540) (Figure 2) or from any cause (41.6% vs. 40.9% vs. 31.4%; *p* = 0.301) (Figure 3) according to baseline potassium levels. No differences were found either when patients were stratified by quartiles according to baseline potassium levels, either in the short or long term, for readmissions for HF (*p* = 0.52), mortality from HF (*p* = 0.75) or all-cause mortality (*p* = 0.73).

No significant differences in the distribution of causes of death were observed between the groups (Table S4). Among non-cardiac deaths, the most frequent causes were infections and malignancy. Furthermore, none of the deaths were due to an abnormality in potassium levels. Among patients who were readmitted for heart failure, the mean number of hospitalisations during follow-up was 2.18 ± 1.97. No significant differences were observed in the number of heart failure hospitalisations between the groups (1.82 ± 1.47 in the hypokalaemia group vs. 2.22 ± 2.03 in the normokalaemia group vs. 2.50 ± 2.35 in the hyperkalaemia group; *p* = 0.76). Likewise, the distribution of the causes of heart failure readmission did not differ significantly among the groups (Table S5).

### 3.7. Independent Predictors of Heart Failure Readmission and All-Cause Mortality

In the multivariable analysis, chronic kidney disease (OR 2.84, 95% CI 1.70–4.76; *p* < 0.01), a history of two or more previous heart failure hospitalisations (OR 4.64, 95% CI 2.10–10.28; *p* < 0.01), and anaemia (OR 2.13, 95% CI 1.08–4.18; *p* = 0.03) emerged as independent predictors of heart failure readmission. Independent predictors of all-cause mortality were older age (OR 1.03, 95% CI 1.00–1.06; *p* = 0.03), chronic kidney disease (OR 2.10, 95% CI 1.13–3.91; *p* = 0.02), a history of two or more previous heart failure hospitalisations (OR 4.69, 95% CI 2.26–9.76; *p* < 0.01), and anaemia (OR 3.06, 95% CI 1.69–5.54; *p* < 0.01). Baseline serum potassium levels were not independently associated with either heart failure readmission or all-cause mortality (Tables S6 and S7).

## 4. Discussion

Potassium abnormalities are a common and clinically relevant issue in the management of patients with HFrEF, as they may limit the use of guideline-directed medical therapy (GDMT), potentially leading to worse clinical outcomes. Although several clinical trials and observational studies have investigated the prevalence of these abnormalities, particularly hyperkalaemia, their clinical and prognostic implications have not been extensively explored until recent years.

The present study evaluated the prognostic impact of baseline potassium abnormalities in a cohort of 409 patients with HFrEF managed in routine clinical practice. In addition, we assessed patients’ clinical characteristics, optimisation of medical therapy, ventricular reverse remodeling, and clinical outcomes according to baseline serum potassium levels.

In our cohort, the prevalence of potassium abnormalities was 10.8%, which is slightly lower than that reported in previous clinical trials and observational studies involving patients with HFrEF. This difference may be explained by the heterogeneity in the potassium thresholds used across studies [12], the use of therapies such as SGLT2i, which are associated with a lower risk of potassium disturbances and were not included in previous studies [13], or the predominance of ambulatory patients in our cohort, who are less likely to develop potassium abnormalities than patients hospitalised for HF. Furthermore, it should be noted that serum potassium levels were assessed only at two predefined time points during follow-up, without information on their intermediate evolution. Consequently, we were unable to determine whether clinically relevant fluctuations occurred throughout follow-up, an issue for which previous studies have reported inconsistent findings [13,14,15].

Regarding factors associated with potassium abnormalities, previous studies have identified the use of guideline-directed medical therapy and diuretics [3,4,5], diabetes mellitus, CKD [8], worse NYHA functional class, and prior hospitalisations [2] as important predictors. Sex-related differences remain less consistent, although some reports suggest that male sex is associated with a higher risk of hyperkalaemia [8], whereas hypokalaemia appears to be more common among women [2].

In our cohort, patients with baseline hypokalaemia had a higher prevalence of diabetes mellitus, which may be explained by insulin therapy, as well as by more advanced heart disease requiring more frequent use of intensive diuretic therapies and other medications, as discussed below, and a greater proportion of patients with two or more previous heart failure hospitalisations. In addition, there was a trend toward a higher proportion of women in this group, although this did not reach statistical significance, probably owing to the limited sample size. Despite this trend, no prognostic differences were observed among women, consistent with previous findings in patients with HFrEF [16].

Regarding baseline clinical status, ≥2 previous HF hospitalisations is a well-established marker of poor prognosis. In our study, consistent with previous reports [17], this characteristic was associated with a trend toward lower serum potassium levels, which may indicate more advanced disease and greater exposure to diuretic therapy during episodes of heart failure decompensation. Indeed, the multivariable logistic regression model showed that ≥2 previous HF hospitalisations was an independent predictor of heart failure readmission and all-cause mortality. However, no differences in NYHA functional class were observed between groups. This finding may be explained by the characteristic “saw-tooth” clinical course of heart failure, in which periods of clinical deterioration are followed by functional recovery after decompensation [18].

No significant differences were observed in the prevalence of CKD across the different potassium groups, despite the well-established association between these two conditions [2,19]. This finding may be explained by the relatively small number of patients with potassium abnormalities and the resulting limited statistical power to detect differences. At the end of follow-up, a slight decline in renal function was observed across all groups, probably reflecting the natural progression of the disease, the need for diuretic therapy, and the use of guideline-directed medical therapy for HFrEF.

With regard to biomarkers, baseline NT-proBNP and CA-125 levels were comparable across the different potassium groups. These findings are consistent with those reported in previous observational registries [20] and are particularly noteworthy given that patients with baseline hypokalaemia had experienced a greater number of prior heart failure hospitalisations. This observation may be explained by the fact that biomarker measurements were obtained in clinically stable outpatients rather than during episodes of acute decompensation.

Evidence regarding the relationship between serum potassium levels and biomarkers of congestion remains limited. A post hoc analysis of the EVEREST trial [21] reported a modest increase in serum potassium levels during hospitalisation, which was weakly associated with a reduction in BNP concentrations. Likewise, the CHANCE-HF study [22] found no differences in hospitalisations related to potassium abnormalities or in the association between potassium disturbances and CA-125 levels when comparing a conventional decongestion strategy for acute heart failure with a CA-125-guided strategy.

Regarding cardiac remodelling, a global improvement in LVEF was observed both in the overall cohort and in each of the analysed groups, in accordance with the criteria established by the 2021 Universal Definition of HF [12]. This improvement has been associated with a better mid-term prognosis in terms of mortality and hospitalisations [23]. To the best of our knowledge, no previous studies have evaluated the relationship between potassium levels and reverse cardiac remodelling. In our study, only the baseline normokalaemia group showed a significant improvement in reverse remodelling parameters. However, both the hypokalaemia and hyperkalaemia groups showed a favourable trend in these parameters, although without reaching statistical significance, likely due to the small sample size.

Regarding GDMT with prognostic benefit, no significant differences were observed in baseline prescription across the different groups. A trend toward greater use of loop and thiazide diuretics was identified only in patients with hypokalaemia, possibly related to greater previous clinical severity and a higher need for decongestion. At the end of follow-up, an overall reduction in loop diuretic use was observed, except in the baseline hypokalaemia group, which may reflect a more advanced stage of disease and a persistent need for diuretic therapy.

Potassium disorders, particularly hyperkalaemia, represent a well-documented barrier to achieving and maintaining optimal doses of guideline-directed medical therapy, with a potential negative impact on clinical outcomes [8]. Several real-world studies have shown that this target remains far from being achieved [2,3,6]. Thus, up to 36% of patients do not receive β-blockers, 19.5% do not receive RAAS inhibitors, and 70.2% do not receive MRA [24]. In our cohort, medical therapy was reasonably optimised from baseline, with approximately 90% use of RAAS inhibitors, 90% of β-blockers, and 74% of MRAs. The main exception was the low use of SGLT2i (20%), likely because during most of the inclusion period these agents were not yet systematically recommended for patients with HFrEF without diabetes, until the publication of the 2021 ESC guidelines [25]. This favourable therapeutic optimisation was maintained even in patients with baseline hyperkalaemia, despite elevated potassium levels being described as independent predictors of under-titration [26].

Regarding prognostic impact, no significant differences were observed in heart failure readmissions, cardiovascular mortality, or all-cause mortality among the three potassium groups, nor when potassium levels were analysed by quartiles. In the multivariable analysis baseline serum potassium levels were not independently associated with either heart failure readmission or all-cause mortality. These findings contrast with the apparently worse clinical profile observed in patients with baseline hypokalaemia. Similarly, a Spanish registry of HFrEF patients with baseline potassium measurement and one-year follow-up also found no significant differences in rehospitalisation or mortality [1]. Likewise, a European registry did not identify an association between potassium abnormalities and heart failure admissions, although it did report an association with all-cause mortality and a relationship between hypokalaemia and hospitalisations due to cardiovascular causes other than HF [2].

Although potassium abnormalities have classically been associated with a U-shaped relationship with mortality [6], controversy remains as to whether they represent a causal factor for adverse events or merely a marker of greater clinical severity. Available evidence appears to support an association between hypokalaemia and the occurrence of clinical events, whereas the effect of hyperkalaemia may be mediated, at least in part, by the limitations it imposes on the optimisation and maintenance of guideline-directed medical therapy [6,27,28].

Taken together, our findings suggest that baseline potassium abnormalities identify different clinical phenotypes within the HFrEF population, particularly in the case of hypokalaemia. However, no independent association was observed with ventricular remodelling, treatment optimisation, or clinical prognosis during follow-up. These findings reinforce the need for larger prospective studies to clarify the true role of potassium disturbances in the clinical course of patients with HFrEF.

### Limitations

This study has several limitations. First, it is a single-centre, retrospective study, with the inherent biases associated with this design. Although the cohort includes a substantial number of patients, the low prevalence of potassium abnormalities limits the statistical power to detect prognostic differences between groups. The low number of patients with potassium abnormalities have limited our ability to detect clinically relevant prognostic differences. Furthermore, potassium measurements were only available at baseline and at the end of follow-up, with no information on intermediate fluctuations or changes related to medical therapy optimisation. However, by the end of follow-up, no significant differences were observed between the study groups with regard to either the prescription or the dose distribution of the main heart failure therapies.

In addition, the use of SGLT2i was relatively low during the study period, reaching only 38% by the end of follow-up. This is explained by the fact that much of the inclusion period preceded their establishment as standard therapy for HFrEF in the 2021 ESC guidelines, which may limit the assessment of their impact on potassium disturbances.

Finally, no data were available on the use of newer potassium binders, and therefore their potential influence on treatment optimisation or clinical outcomes could not be evaluated.

## 5. Conclusions

In our cohort, patients with HFrEF and baseline hypokalaemia had a higher prevalence of diabetes mellitus and a higher proportion of previous hospital admissions for HF. However, no differences were observed in improvements in LVEF, ventricular remodelling or treatment optimisation with prognostic benefit. Abnormal baseline potassium levels do not appear to be associated with a worse prognosis in terms of readmissions for HF, mortality from HF and all-cause mortality. These findings need to be confirmed in future prospective studies with larger sample sizes.

## Figures and Tables

**Table 1:** Baseline characteristics. CKD: Chronic kidney disease; COPD: Chronic obstructive pulmonary disease. HF: Heart failure; HFrEF: Heart failure with reduced ejection fraction; LBBB: Left bundle branch block; LVEF: Left ventricular ejection fraction. RBBB: Right bundle branch block. ƚ: The *p*-value refers to the differences between the groups based on baseline potassium levels.

	HFrEF *(n = 409)*	Hypokalaemia *(n = 26)*	Normokalaemia *(n = 365)*	Hyperkalaemia *(n = 18)*	*p* ^ƚ^
Sex male	303 (74.1%)	18 (69.2%)	270 (74%)	15 (83.3%)	0.570
Age (years)	69 (55–77)	71 (63–81)	69 (59–76)	68.5 (56.5–78)	0.243
Diabetes mellitus	197 (48.2%)	18 (69.2%)	173 (47.4%)	6 (33.3%)	0.043
Insulin treatment	65 (15.9%)	7 (26.9%)	56 (15.3%)	2 (11.1%)	0.252
Hypertension	274 (67%)	18 (69.2%)	244 (66.8%)	12 (66.7%)	0.969
Dyslipidaemia	254 (62.3%)	20 (76.9%)	224 (61.4%)	10 (58.8%)	0.274
HF de novo	226 (55.3%)	14 (53.8%)	205 (56.2%)	7 (38.9%)	0.351
HF duration (months)	36.3 ± 66.7	36 ± 69.1	35.6 ± 66.5	49.9 ±70.4	0.507
≥2 previous admissions for HF	45 (11%)	7 (25%)	38 (10.5%)	0 (0%)	0.008
LVEF improvement	156 (41.6%)	8 (32%)	143 (43.1%)	5 (27.8%)	0.264
NYHA I-II	328 (80.4%)	21 (80.8%)	292 (80.2%)	15 (83.3%)	0.947
NYHA III-IV	80 (19.6%)	5 (19.2%)	72 (19.8%)	3 (16.7%)	
Ischaemic aetiology	133 (32.5%)	8 (30.8%)	117 (32.1%)	8 (44.4%)	0.538
Valvular aetiology	6 (1.5%)	0 (0%)	6 (1.6%)	0 (0%)	0.693
Tachycardomyopathy	44 (10.8%)	2 (7.7%)	41 (11.2%)	1 (5.6%)	0.655
Cardiotoxicity	15 (3.7%)	1 (3.8%)	14 (3.8%)	0 (0%)	0.699
Oncological aetiology	16 (3.9%)	2 (7.7%)	14 (3.8%)	0 (0%)	0.422
Genetic aetiology	12 (3%)	0 (0%)	11 (3%)	1 (5.6%)	0.541
Idiopathic aetiology	125 (30.6%)	8 (30.8%)	112 (30.7%)	5 (27.8%)	0.268
Atrial fibrillation	214 (52.3%)	16 (61.5%)	189 (51.8%)	9 (50%)	0.617
Obesity	146 (37.3%)	8 (30.8%)	127 (36.6%)	11 (61.1%)	0.086
COPD	75 (18.3%)	7 (26.9%)	63 (17.3%)	5 (27.8%)	0.268
Wide QRS complex	151 (40.6%)	15 (57.7%)	130 (39%)	6 (46.2%)	0.161
RBBB	33 (9.3%)	3 (7.7%)	29 (8.2%)	1 (7.7%)	<0.001
LBBB	117 (32.7%)	12 (46.2%)	101 (32%)	4 (30.8%)	
CKD	170 (41.6%)	16 (61.5%)	145 (39.7%)	9 (50%)	0.070
Grade IIIa	55 (13.5%)	5 (19.2%)	47 (12.9%)	3 (16.7%)	0.247
Grade IIIb	66 (16.1%)	5 (19.2%)	57 (15.6%)	4 (22.2%)	
Grade IV	35 (8.6%)	6 (23.1%)	29 (8%)	0 (0%)	
Grade V	14 (3.4%)	0 (0%)	12 (3.3%)	2 (11.1%)	
Anaemia	130 (31.8%)	12 (46.2%)	112 (30.7%)	6 (33.3%)	0.259

**Table 2:** **Treatment characteristics, echocardiographic and laboratory parameters by group at the baseline and end of follow-up.** GFR: Glomerular filtration rate; Hb: Haemoglobin; HFrEF: Heart failure with reduced ejection fraction; LVEF: Left ventricular ejection fraction; LVEDD: Left ventricular end-diastolic diameter. LVESD: Left ventricular end-systolic diameter; LVEDV: Left ventricular end-diastolic volume; LVESV: Left ventricular end-systolic volume; MR: Mitral regurgitation. ƚ: The *p*-value refers to the differences between the groups based on baseline potassium levels.

	Baseline					Final				
	HFrEF (n = 409)	Hypokalaemia (n = 26)	Normokalaemia (n = 365)	Hyperkalaemia (n = 18)	*p* ^ƚ^	HFrEF (n = 409)	Hypokalaemia (n = 26)	Normokalaemia (n = 365)	Hyperkalaemia (n = 18)	*p* ^ƚ^
LVEF (%)	30 (26–35)	30.5 (25–36)	30 (26–35)	32 (28–35)	0.800	37 (30–51.8)	35 (40–48.5)	38 (30–52.5)	35 (30–45.8)	0.504
LVEDD (mm)	62 (57–68)	64 (56–69.5)	62 (57–68)	61 (56–66)	0.885	58 (53–63)	61.5 (55–65)	58 (53–63)	56 (51–62)	0.408
LVESD (mm)	52 (47–59)	53 (49–60)	52 (47–59)	50 (49–67)	0.829	45 (38–52)	46.5 (40.5–52.5)	44.5 (37.3–52)	51 (37–55.5)	0.594
LVEDV (mL)	153 (118–188)	136 (108–172)	155 (118–198)	154 (118–197)	0.378	124 (91–167)	123 (76–171)	124 (94–167)	110 (90–133)	0.729
LVESV (mL)	105 (78–137)	92 (76–124)	106 (79–141)	100 (78–127)	0.507	73 (47–108)	74 (40–93)	73.5 (47.8–110)	67 (50–88)	0.721
Severe MR (%)	44 (10.9%)	2 (7.7%)	39 (10.9%)	3 (16.7%)	0.641	21 (5.7%)	1 (4.3%)	20 (6.1%)	0 (0%)	0.549
Creatinine (mg/dL)	1.1 (0.9–1.4)	1.2 (0.8–1.9)	1.1 (0.9–1.4)	1.1 (1–1.5)	0.466	1.2 (1–1.6)	1.4 (1–1.8)	1.2 (1–1.6)	1.3 (1.1–1.5)	0.083
GFR (mL/min)	68 (47–86)	59 (34–94)	69 (48–86)	62 (48–78)	0.685	59 (40–78)	46 (29–67)	61 (40–80)	52 (46–69)	0.089
Hb (g/dL)	13.7 (12–15.2)	13 (11.2–14.3)	13.7 (12.1–15.3)	13.6 (11.3–15.6)	0.139	13.6 (12.1–15.3)	13.1 (11.5–13.9)	13.6 (12.1–15.4)	14.3 (12.4–15.5)	0.222
Sodium (mEq/L)	140 (137–142)	140 (138–142)	140 (137–142)	139 (136–142)	0.725	140 (138–142)	138 (137–142)	140 (138–142)	139.5 (138–141)	0.262
Potassium (mEq/L)	4.4 (4–4.7)	3.2 (3.1–3.3)	4.4 (4–4.7)	5.7 (5.5–5.8)	<0.001	4.5 (4.2–4.9)	4.7 (3.9–5.2)	4.5 (4.2–4.9)	4.9 (4.2–5.4)	0.290
CA-125 (U/mL)	21.8 (9.5–69)	96 (10–145)	19.7 (9.3–66)	22.4 (10–37.9)	0.227	10.1 (6.2–21)	10.1 (7.3–134.6)	10.1 (6.2–24.6)	10.3 (5.5–16.9)	0.940
NT-proBNP (pg/mL)	4822 (2081–10,964)	5896 (1837–10,783)	4771 (2106–11,237)	3762 (1459–13,717)	0.854	1934 (611–6317)	2201 (749–8592)	1905 (616–6247)	3346 (270–5104)	0.661

## Data Availability

No material from other sources was reproduced in this manuscript. All content is original. The data presented in this study are available on request from the corresponding author. The data are not publicly available due to privacy and ethical restrictions.

## Supplementary Materials

The following supporting information can be downloaded at: https://www.mdpi.com/article/10.3390/medsci14030402/s1, Table S1: Dosage guidelines for pharmacological treatment in patients with heart failure with reduced ejection fraction. ARNI: Angiotensin Receptor-Neprilysin Inhibitor; MRA: Mineralocorticoid receptor antagonist. Table S2. Treatment characteristics at baseline and at the end of follow-up. ACEi: Angiotensin-converting enzyme inhibitors; ARB: angiotensin receptor blocker; ARNI: Angiotensin Receptor-Neprilysin Inhibitor; CRT: Cardiac resynchronisation therapy; HFrEF: Heart failure with reduced ejection fraction; ICD: implantable cardioverter-defibrillator; MRA: Mineralocorticoid receptor antagonist; PVM: Percutaneous mitral valve treatment; SGLT2i: Sodium-glucose cotransporter 2 inhibitors. Table S3. Drug dose titration in HFrEF according to potassium levels at the baseline and end of follow-up. ARNI: Angiotensin Receptor-Neprilysin Inhibitor; MRA: mineralocorticoid receptor antagonist. Table S4. Causes of death according to potassium levels at the baseline. Table S5. Causes of heart failure readmission according to potassium levels at the baseline. Table S6. Univariate logistic regression analysis and multivariable logistic regression model to identify independent predictors of heart failure readmission. Predictors of heart failure readmissions. COPD: Chronic obstructive pulmonary disease. DM: Diabetes mellitus. HF: Heart failure. HyperK: Hyperkalemia. HypoK: Hypokalemia. NormoK: Normokalemia. OR: Odds Ratio. Table S7. Univariate logistic regression analysis and multivariable logistic regression model to identify independent predictors of all-cause mortality. Predictors of all-cause mortality. COPD: Chronic obstructive pulmonary disease. DM: Diabetes mellitus. HF: Heart failure. HyperK: Hyperkalemia. HypoK: Hypokalemia. NormoK: Normokalemia. OR: Odds Ratio.

## Abbreviations

The following abbreviations are used in this manuscript:

ACEi	Angiotensin-Converting Enzyme inhibitors
ARB	Angiotensin Receptor Blockers
ARNI	Angiotensin Receptor–Neprilysin Inhibitor
CA-125	Carbohydrate Antigen 125
CKD	Chronic Kidney Disease
COPD	Chronic Obstructive Pulmonary Disease
CRT	Cardiac Resynchronisation Therapy
DM	Diabetes Mellitus
GDMT	Guideline-Directed Medical Therapy
GFR	Glomerular Filtration Rate
HF	Heart Failure
HFrEF	Heart Failure with reduced Ejection Fraction
ICD	Implantable Cardioverter-Defibrillator
LBBB	Left Bundle Branch Block
LVEDD	Left Ventricular End-Diastolic Diameter
LVEDV	Left Ventricular End-Diastolic Volume
LVEF	Left Ventricular Ejection Fraction
LVESD	Left Ventricular End-Systolic Diameter
LVESV	Left Ventricular End-Systolic Volume
MR	Mitral Regurgitation
MRA	Mineralocorticoid Receptor Antagonists
NT-proBNP	N-Terminal pro-Brain Natriuretic Peptide
NYHA	New York Heart Association
RAAS	Renin–Angiotensin–Aldosterone System
SGLT2i	Sodium–Glucose Cotransporter 2 inhibitors
WHF	Worsening Heart Failure
