# Supplementary material for: Prognostic Impact of Baseline Potassium Abnormalities in Patients with Heart Failure with Reduced Ejection Fraction: A Real-World Cohort Study
Source: Med Sci (Basel). 2026 Jul 18;14(3):402. doi: 10.3390/medsci14030402 (PMC13414006; doi:10.3390/medsci14030402)
Supplement: Supplementary file 1 [file medsci-14-00402-s001.zip › medsci-4437668-supplementary.pdf]

**ARNI**	**Low dose**	≤ 100 mg / 24 hours
	**Medium dose**	100-200 mg / 24 hours
	**High dose**	> 200 mg / 24 hours
**β-blockers**	**Low dose**	Bisoprolol ≤ 2.5 mg / 24 hours; carvedilol < 1.,5 mg / 12 hours; nebivolol ≤ 2.5 mg / 24 hours; atenolol < 50 mg / 24 hours
	**Medium dose**	Bisoprolol 2.5-5 mg / 24 hours; carvedilol 12.5-25 mg / 12 hours; nebivolol 2.5-5 mg / 24 hours; atenolol 50-75 mg / 24 hours
	**High dose**	Bisoprolol > 5 mg / 24 hours; carvedilol > 25 mg / 12 hours; nebivolol > 5 mg / 24 hours; atenolol > 75 mg / 24 hours
**MRA**	**Low dose**	Eplerenone ≤ 25 mg / 24 hours; spironolactone ≤ 25 mg / 24 hours
	**High dose**	Eplerenone ≤ 25 mg / 24 hours; spironolactone ≤ 25 mg / 24 hours
**Ivabradine**	**Low dose**	≤ 5 mg / 12 hours
	**High dose**	> 5 mg / 12 hours
**Loop diuretics**	**Low dose**	Furosemide ≤ 120 mg / 24 hours (or another loop diuretic at an equivalent dose)
	**High dose**	Furosemide > 120 mg / 24 hours (or another loop diuretic at an equivalent dose)
**Thiazides**	**Low dose**	Hydrochlorothiazide ≤ 25 mg/24 hours - Chlorthalidone ≤ 25 mg/24 hours
	**High dose**	Hydrochlorothiazide > 25 mg/24 hours - Chlorthalidone > 25 mg/24 hours

Table S1. Dosage guidelines for pharmacological treatment in patients with heart failure with reduced ejection fraction. ARNI: Angiotensin Receptor-Neprilysin Inhibitor; MRA: Mineralocorticoid receptor antagonist.

	BASELINE					FINAL				
	HFrEF (n=409)	Hypokalaemia (n=26)	Normokalaemia (n=365)	Hyperkalaemia (n=18)	p	HFrEF (n=409)	Hypokalaemia (n=26)	Normokalaemia (n=365)	Hyperkalaemia (n=18)	p
**ACEi/ARB**	234 (57.2%)	16 (61.5%)	218 (58.1%)	6 (33.3%)	0.105	127 (31.1%)	7 (26.9%)	116 (31.8%)	4 (22.2%)	0.621
**ARNI**	140 (34.2%)	8 (30.8%)	124 (34%)	8 (44.4%)	0.612	243 (59.4%)	17 (65.4%)	213 (58.4%)	13 (72.2%)	0.411
**β-Blockers**	374 (91.4%)	23 (88.5%)	334 (91.5%)	17 (94.4%)	0.777	357 (88.5%)	22 (84.6%)	318 (87.1%)	17 (94.4%)	0.604
**Loop diuretic**	328 (80.2%)	23 (88.5%)	291 (79.7%)	14 (77.8%)	0.539	293 (71.6%)	25 (96.2%)	257 (70.4%)	11 (61.1%)	0.011
**Thiazides**	77 (18.8%)	9 (34.6%)	64 (17.5%)	4 (22.2%)	0.092	78 (19.1%)	8 (30.8%)	69 (18.9%)	1 (5.6%)	0.108
**MRA**	301 (73.6%)	18 (69.2%)	269 (73.7%)	14 (77.8%)	0.811	286 (69.9%)	20 (76.9%)	252 (69%)	14 (77.8%)	0.530
**Ivabradine**	88 (21.5%)	3 (11.5%)	81 (22.2%)	4 (22.2%)	0.441	78 (19.1%)	4 (15.4%)	71 (19.5%)	3 (16.7%)	0.848
**Digoxin**	48 (11.7%)	3 (11.5%)	44 (12.1%)	1 (5.6%)	0.704	50 (12.2%)	5 (19.2%)	43 (11.8%)	2 (11.1%)	0.528
**SGLT2i**	76 (18.6%)	5 (19.2%)	69 (18.9%)	2 (11.1%)	0.706	154 (37.7%)	11 (42.3%)	136 (37.3%)	7 (38.9%)	0.871
**ICD**	37 (9%)	3 (11.5%)	31 (8.5%)	3 (16.7%)	0.449	73 (17.8%)	4 (15.4%)	64 (17.5%)	5 (27.8%)	0.511
**CRT**	19 (4.6%)	1 (3.8%)	17 (4.7%)	1 (5.6%)	0.965	69 (16.9%)	2 (7.7%)	63 (17.3%)	4 (22.2%)	0.374
**PVM**	1 (0.2%)	0 (0%)	1 (0.2%)	0 (0%)	0.941	15 (3.7%)	0 (0%)	14 (3.8%)	1 (5.6%)	0.549
**Urgent haemodialysis**	-	-	-	-	-	9 (2.2%)	1 (3.8%)	8 (2.2%)	0 (0%)	0.693

Table S2. Treatment characteristics at baseline and at the end of follow-up. ACEi: Angiotensin-converting enzyme inhibitors; ARB: angiotensin receptor blocker; ARNI: Angiotensin Receptor-Neprilysin Inhibitor; CRT: Cardiac resynchronisation therapy; HFrEF: Heart failure with reduced ejection fraction; ICD: implantable cardioverter-defibrillator; MRA: Mineralocorticoid receptor antagonist; PVM: Percutaneous mitral valve treatment; SGLT2i: Sodium-glucose cotransporter 2 inhibitors.

		Hypokalaemia			Normokalaemia			Hyperkalaemia		
		Baseline	Final	P	Baseline	Final	p	Baseline	Final	p
ARNI (%)	Low dose	8 (100%)	9 (52.9%)	0.374	84 (67.7%)	85 (39.9%)	<0.001	3 (37.5%)	5 (38.5%)	0.285
	Medium dose	0 (0%)	3 (17.6%)		30 (24.2%)	59 (27.7%)		5 (62.5%)	3 (23.1%)	
	High dose	0 (0%)	5 (29.4%)		10 (8.1%)	69 (32.4%)		0 (0%)	5 (38.5%)	
β-Blocker (%)	Low dose	15 (65.2%)	9 (40.9%)	0.031	198 (59.3%)	159 (50%)	<0.001	8 (47.1%)	7 (41.2%)	0.431
	Medium dose	7 (30.4%)	10 (45.5%)		106 (31.7%)	104 (32.7%)		7 (41.2%)	7 (41.2%)	
	High dose	1 (4.3%)	3 (13.6%)		30 (9%)	55 (17.3%)		2 (11.8%)	3 (17.6%)	
MRA (%)	Low dose	17 (94.4%)	17 (85%)	0.500	256 (95.2%)	227 (90.1%)	0.041	13 (92.9%)	11 (78.6%)	0.500
	High dose	1 (5.6%)	3 (15%)		13 (4.8%)	25 (9.9%)		1 (7.1%)	3 (21.4%)	
Ivabradine (%)	Low dose	1 (33%)	1 (25%)	-	60 (74.1%)	54 (76.1%)	1.000	3 (75%)	2 (66.7%)	1.000
	High dose	2 (66.7%)	3 (75%)		21 (25.9%)	17 (23.9%)		1 (25%)	1 (33.3%)	
Loop diuretics (%)	Low dose	23 (100%)	24 (96%)	1.000	283 (97.3%)	244 (94.9%)	0.302	14 (100%)	10 (90.9%)	-
	High dose	0 (0%)	1 (4%)		8 (2.7%)	13 (5.1%)		0 (0%)	1 (9.1%)	
Thiazides (%)	Low dose	7 (77.8%)	7 (87.5%)	1.000	54 (84.4%)	52 (75.4%)	1.000	3 (75%)	0 (0%)	-
	High dose	2 (22.2%)	1 (12.5%)		10 (15.6%)	17 (24.6%)		1 (25%)	1 (100%)	
Monotherapy – Dual therapy		11 (42.3%)	8 (30.8%)	0.250	115 (31.5%)	115 (31.5%)	1.000	6 (33.3%)	4 (22.2%)	0.625
Triple–quadruple therapy		15 (57.7%)	18 (69.2%)		250 (68.5%)	250 (68.5%)		12 (66.7%)	14 (77.8%)	

Table S3. Drug dose titration in HFrEF according to potassium levels at the baseline and end of follow-up. ARNI: Angiotensin Receptor-Neprilysin Inhibitor; MRA: mineralocorticoid receptor antagonist.

	Hypokalaemia (10 deaths)	Normokalaemia (110 deaths)	Hyperkalaemia (5 deaths)	p
**Heart failure related**	6 (60%)	55 (50%)	3 (60%)	0.898
**Sudden cardiac death**	1 (10%)	25 (22.7%)	1 (20%)	
**Non-cardiac related**	3 (30%)	30 (27.3%)	1 (20%)	

Table S4. Causes of death according to potassium levels at the baseline.

	Hypokalaemia (10 readmissions)	Normokalaemia (110 deaths)	Hyperkalaemia (5 deaths)	p
**Idiopathic**	3 (30%)	58 (46.8%)	3 (50%)	0.852
**Poor treatment adherence**	1 (10%)	6 (4.8%)	0 (0%)	
**Infection**	3 (30%)	23 (18.5%)	2 (33.3%)	
**Anaemia**	0 (0%)	9 (7.3%)	0 (0%)	
**Other**	3 (30%)	28 (22.6%)	1 (16.7%)	

Table S5. Causes of heart failure readmission according to potassium levels at the baseline.

	UNIVARIATE ANALYSIS			MULTIVARIATE ANALYSIS		
	OR	95% CI	p-value	OR	95% CI	p-value
**Age**	1.04	1.02-1.05	<0.01	1.00	0.98-1.02	0.75
**Sex male**	1.24	0.78-1.95	0.36			
**DM**	1.23	0.82-1.85	0.31			
**COPD**	1.85	1.11-3.08	<0.05	1.59	0.89-2.83	0.12
**CKD**	5.54	3.59-8.67	<0.01	2.84	1.70-4.76	<0.01
**≥2 previous admissions for HF**	7.88	3.91-17.3	<0.01	4.64	2.10-10.28	<0.01
**Ischaemic ethiology**	1.86	1.21-2.85	<0.05	1.11	0.67-1.84	0.68
**HypoK vs NormoK**	1.43	0.62-3.22	0.40			
**HyperK vs NormoK**	0.91	0.31-2.39	0.85			
**Anaemia**	5.08	3.25-8.01	<0.01	2.13	1.08-4.18	0.03

Table S6. Univariate logistic regression analysis and multivariable logistic regression model to identify independent predictors of heart failure readmission. **Predictors of heart failure readmissions.** COPD: Chronic obstructive pulmonary disease. DM: Diabetes mellitus. HF: Heart failure. HyperK: Hyperkalemia. HypoK: Hypokalemia. NormoK: Normokalemia. OR: Odds Ratio

	UNIVARIATE ANALYSIS			MULTIVARIATE ANALYSIS		
	OR	95% CI	p-value	OR	95% CI	p-value
**Age**	1.06	1.03-1.08	<0.01	1.03	1.00-1.06	0.03
**Sex male**	0.98	0.57-1.66	0.95			
**DM**	1.14	0.71-1.83	0.59			
**COPD**	2.09	1.19-3.60	<0.01	1.84	0.98-3.44	0.06
**CKD**	5.63	3.37-9.65	<0.01	2.10	1.13-3.91	0.02
**≥2 previous admissions for HF**	7.23	3.78-14.1	<0.01	4.69	2.26-9.76	<0.01
**Ischaemic ethiology**	2.43	.50-3.95	<0.05	1.48	0.85-2.60	0.17
**HypoK vs NormoK**	1.42	0.54-3.39	0.45			
**HyperK vs NormoK**	1.04	0.29-3.01	0.94			
**Anaemia**	5.08	3.25-8.01	<0.01	3.06	1.69-5.54	<0.01

Table S7. Univariate logistic regression analysis and multivariable logistic regression model to identify independent predictors of all-cause mortality. **Predictors of all-cause mortality.** COPD: Chronic obstructive pulmonary disease. DM: Diabetes mellitus. HF: Heart failure. HyperK: Hyperkalemia. HypoK: Hypokalemia. NormoK: Normokalemia. OR: Odds Ratio
